# Fuzheng Jiedu Decoction Induces Apoptosis and Enhances Cisplatin Efficacy in Ovarian Cancer Cells *In Vitro* and *In Vivo* through Inhibiting the PI3K/AKT/mTOR/NF-*κ*B Signaling Pathway

**DOI:** 10.1155/2022/5739909

**Published:** 2022-03-02

**Authors:** Huadi Yang, Hui Li, Shenyi Lu, Shuangshuang Shan, Yong Guo

**Affiliations:** ^1^Department of Gynecology and Obstetrics, The First Affiliated Hospital of Zhejiang Chinese Medical University (Zhejiang Provincial Hospital of Traditional Chinese Medicine), Hangzhou, Zhejiang 310006, China; ^2^Department of Oncology, The First Affiliated Hospital of Zhejiang Chinese Medical University (Zhejiang Provincial Hospital of Traditional Chinese Medicine), Hangzhou, Zhejiang 310006, China

## Abstract

**Objectives:**

This study is aimed at investigating the anticancer activity of Fuzheng Jiedu decoction (FJD) alone or in combination with cisplatin in ovarian cancer (OC) models, as well as its underlying mechanisms of action.

**Methods:**

The anticancer activities of FJD, cisplatin, and the combination of the PI3K inhibitor (LY294002, LY) or activator (IGF-1) were evaluated in OC cell lines *in vitro* and in a SKOV3 xenograft mouse model *in vivo*. The cell proliferation and invasion ability were measured using MTT, EdU, and transwell assays, respectively. The cell apoptosis was examined by flow cytometry and JC-1 assays. The expression levels of the Bcl-2 family and the PI3K/AKT/mTOR/NF-*κ*B pathway-related proteins were analyzed by Western blot.

**Results:**

The *in vivo* and *in vitro* studies showed that FJD administration could significantly inhibit cell proliferation and promote cell apoptosis in two OC cell lines SKOV3 and 3AO and partially decreased the tumor volumes and weights. In addition, FJD could significantly downregulate the protein levels of p-PI3K/PI3K, p-AKT/AKT, p-mTOR/mTOR, NF-*κ*B, p38, and Bcl-2 and upregulate the Bax, Cyt-C, and cleaved caspase-3 in OC tumor tissues and cells. FJD cotreatment increased the efficacy of cisplatin, including inhibiting OC cell proliferation and invasion, promoting cell apoptosis, and inhibiting the PI3K/AKT/mTOR signaling pathway, while this enhancement was suppressed by IGF-1. Similarly, LY also enhanced the anticancer efficacy of cisplatin.

**Conclusions:**

This study indicated that FJD could improve the efficacy of cisplatin by inhibiting the PI3K/AKT/mTOR/NF-*κ*B signaling pathway. It is suggested that FJD may be a valuable adjuvant drug for the treatment of OC.

## 1. Introduction

Ovarian cancer (OC) is the second most common gynecological malignancy and ranks first in mortality among gynecological tumors [1]. According to the global cancer statistics report in 2018, there are 295,414 new cases of OC worldwide, and the number of death cases reaches 184,800 [2]. More importantly, the incidence and prevalence of OC continue to rise annually. Although important progresses have been made in OC diagnosis as early as possible, recently, OC patients diagnosed at a late stage still account for a certain part. Even if there are many emerging and effective drugs, the prognosis of OC remains unsatisfactory. Women who were diagnosed with OC between 2000 and 2007 have an average survival of 5 years in Europe, with a very poor survival rate until now [3]. Therefore, the development of new treatment methods is very important for the clinical effective treatment of OC.

Studies have shown that the PI3K/AKT/mTOR signaling pathway is a main cancer-promoting factor [4] and participates in a variety of physiological functions and disease processes such as cell survival, angiogenesis, proliferation, and metabolism [5–7]. It has been reported that the PI3K/AKT/mTOR/NF-*κ*B signaling pathway is an attractive target for the treatment of invasive cancer [8]. PI3K is a vital oncogenic protein related to enzyme activity and acts on cancer progression [9]. Since the aberrant activation of the PI3K/AKT/mTOR signaling pathway often happens in most of the human cancers [10, 11], targeting this pathway may be a valuable strategy for the treatment of cancer. It is worth noting that the PI3K/AKT/mTOR signaling pathway is activated in about 70% of ovarian cancers [12], which suggested that one or more correlated pathways are affected [13]. Furthermore, it has been found that the PI3K activation leads to the increased survival and chemoresistance of cancer cells [14]. Therefore, targeting the PI3K pathway could be a promising method for the treatment of ovarian cancer. Although PI3K inhibitors have made great contributions in inhibiting tumors, drug resistance or side effects limit their therapeutic effects. Therefore, it is necessary to further develop new therapeutic methods combined with chemotherapy to improve the antitumor efficacy.

Fuzheng Jiedu decoction (FJD) is a traditional Chinese medicine (TCM) compound prescription that is composed of *Pseudostellaria heterophylla* (Miq.) Pax, *Atractylodes macrocephala* Koidz, *Poria cocos* (Schw.) Wolf, *Actinidia chinensis* Planch, and *Polygonum cuspidatum* Sieb. et Zucc. It is reported to regulate the human immune function and tumor microenvironment to exert antitumor efficacy through the multitarget, multilevel, and multichannel synergy [15, 16]. Previous studies revealed that the mechanisms of FJD in treating malignant tumors include enhancing and regulating the body's immune ability, regulating the balance of the tumor extracellular matrix, inhibiting tumor cell proliferation and angiogenesis, inducing tumor cell apoptosis, and preventing tumor cell genes from mutation [17–20]. However, the regulation mechanisms of FJD in OC have not been elucidated. Thus, we hypothesized that FJD has anticancer potential on OC, and this may be achieved by intervening the PI3K/AKT/mTOR/NF-*κ*B signaling pathway. Cisplatin is a platinum-based drug widely applied in cancer chemotherapy, and coadministration of cisplatin with TCM has been reported to achieve effect enhancement and toxicity reduction in cancers [17]. Therefore, this study focuses on the anticancer activities of FJD alone and in combination with cisplatin in OC cells *in vivo* and *in vitro*, launching a basis for the clinical application of FJD.

## 2. Materials and Methods

### 2.1. Preparation of Fuzheng Jiedu Decoction (FJD)

The FJD was composed of five herbs: *Pseudostellaria heterophylla* (Miq.) Pax, *Atractylodes macrocephala* Koidz, *Poria cocos* (Schw.) Wolf, *Actinidia chinensis* Planch, and *Polygonum cuspidatum* Sieb. et Zucc. These herbs were mixed and crushed according to the weight ratio of 15 g, 10 g, 10 g, 15 g, and 15 g. After being soaked in 500 ml distilled water for 30 min, the herbs were boiled and extracted twice; the extracted solution was then concentrated to a final solution of 0.262, 0.524, and 1.048 g/ml, respectively. The obtained FJD was stored at -4°C for the subsequent experiment. The chemical components of FJD were also analyzed using the UPLC-Q/TOF-MS method.

### 2.2. Animals

Healthy SPF Balb/c nude mice (female, 4-6 weeks old, bodyweight 18-25 g) were purchased from Shanghai SLAC Laboratory Animal Co., Ltd. (license no. SCXK (Hu) 2013-0018; Shanghai, China). The follow-up experiment was carried out after one week of adaptive feeding. All animal experiments were approved by the Animal Ethics Committee (Hangzhou Eyong Biotechnological Co., Ltd., license no. SYXK (Zhe) 2020-0024).

### 2.3. Tumor Xenograft Assay

The mice were injected with 0.1 ml of SKOV3 cell suspension (1 × 10^7^ cells/mL) [21]. The subcutaneous tumor grew to 100 mm^3^ about two weeks later indicating that the tumor xenograft model was successfully established. The model mice were randomly divided into different groups (five mice in each group): model group, FJD-L, FJD-M, and FJD-H (low, medium, and high doses of FJD were 5.24, 10.48, and 20.96 g/kg/d, respectively) groups, Cis group (cisplatin, 3 mg/kg/3 d), Cis+FJD-H group (cisplatin, 3 mg/kg/3 d+FJD-H, 20.96 g/kg/d), Cis+LY group (cisplatin, 3 mg/kg/3 d+PI3K inhibitor, LY294002, 40 mg/kg/d), and Cis+FJD-H+IGF1 group (cisplatin, 3 mg/kg/3 d+FJD-H, 20.96 g/kg/d+PI3K activator, IGF-1, 1.6 *μ*g/kg/d). The mice were administered for 20 days.

### 2.4. Tumor Volume and Weight Detection

Tumor volume was measured 2 d/time. After 20 days of administration, the mice were sacrificed. Tumor tissues were extracted, photographed, and weighed. The appropriate dosage of FJD in the follow-up experiment was chosen according to the results of the tumor inhibition rate with different dosages of FJD administration.

### 2.5. HE Staining

Ovarian cancer tissues in nude mice were extracted and placed in 4% paraformaldehyde. The tumor tissues were further made into paraffin sections. Paraffin sections were roasted in 60 degrees oven until the water was dried and wax dried, then they were removed and stored. They were stained with hematoxylin and eosin for 3 min, respectively, and it was observed under the microscope after mounting.

### 2.6. Cell Culture and Treatment

The OC cell lines SKOV3 and 3AO were bought from the iCell Bioscience (Shanghai, China). Culture cells containing 10% defined FBS using RPMI-1640 medium (Gibco, Auckland, N.Z.). The tumor cells were cultured in a 5% of CO_2_ incubator at 37°C. SKOV3 and 3AO cells were divided into different groups: control group (10% control serum), LY group (LY294002, 100 ng/ml), FJD-H group (20% FJD-containing serum), FJD-H+IGF1 group (20% FJD-containing serum, IGF-1 100 ng/ml), Cis group (cisplatin, 4800 ng/ml), Cis+FJD-H group (cisplatin 4800 ng/ml, 20% FJD contained serum), Cis+LY group (cisplatin 4800 ng/ml, LY294002, 100 ng/ml), and Cis+FJD-H+IGF1 group (cisplatin 4800 ng/ml, 20% FJD-containing serum, and IGF-1 100 ng/ml).

### 2.7. MTT Assay

SKOV3 and 3AO cells which are in a logarithmic growth phase were, respectively, planted in a 6-well plate, and the culture plates were precultivated in an incubator for 24 h; digest and centrifuge the cells in the six-well plate after administration for 24, 48, and 96 h. Then, digest and centrifuge cells in the six-well plate, wash them once with PBS, resuspend them with medium after centrifugation, and add cells to the 96-well plate. Add 20 *μ*l MTT (5 g/l) and continue to incubate for 4 h; then, discard the culture medium, add 200 *μ*l/well of DMSO, shake for 10 min until the crystals are dissolved, measure the absorbance at 490 nm wavelength, and calculate the cell survival rate. Three replicate wells were measured in parallel for each group.

### 2.8. EdU Assay

After SKOV3 and 3AO cells were treated with certain drugs, 500 *μ*l diluted EdU solution was added to each well and incubated at 37°C for another 4 h. The cells were then fixed and permeabilized with ethanol (95%) and Triton X-100 for 15 and 10 min, respectively. After rinsing with PBS three times, click reaction solution and Hoechst 33342 solution were successively added and incubated in the dark for 30 min. After sealing, the stained cells were observed under a fluorescence microscope.

### 2.9. Transwell Assay

Dilute Matrigel with serum-free DMEM high-glucose medium to 3 : 1, the transwell chambers were coated with 30 *μ*l Matrigel dilution and then incubated overnight at 4°C. SKOV3 and 3AO cells were treated with drugs for 24 h and then added to the upper chamber. The incubator conditions were maintained in the gas environment of 5% CO_2_ at 37°C. The cells were fixed with paraformaldehyde and stained with crystal violet for 30 min, and the lower ventricular cells were counted for 3 parallel.

### 2.10. JC-1 Assay

The SKOV3 and 3AO cells were collected (3-5 × 10^5^ cells per 6-well plate); then, the cells were cleaned with PBS and added with 1 mL incomplete medium. Add 1 ml diluted JC-1 staining solution, and cells were incubated in an incubator at 37°C for 20 min away from light. After incubation, the supernatant was aspirated, and the cells were digested with trypsin before centrifuging to retain the precipitation. Then, the cells were suspended with JC-1 staining buffer, and the results were analyzed by flow cytometry.

### 2.11. Flow Cytometry

Cells that are in the logarithmic growth phase were planted in a 6-well plate. The cells were counted, and the concentration was adjusted to 1 × 10^6^ cells/ml. Next, the cells were mixed with 500 *μ*l buffer, and the precipitate obtained by centrifugation was resuspended with buffer. Subsequently, 5 *μ*l Annexin V-FITC and PI were added and mixed and kept in the dark for 30 min. The apoptosis rate was detected.

### 2.12. Western Blotting Analysis

The OC tumor tissue and cells were extracted to obtain the protein, and the protein was quantified by using the BCA kit. The 50 *μ*g protein was loaded quantitatively and separated by SDS-PAGE with 10% separation gel. Electrophoresis conditions were as follows: 80 V (30 min) and then 120 V; electrophoretic transfer condition was as follows: 90 min, 200 mA, and transfer protein to the PVDF membrane; it was then sealed for 2 h in skim milk powder solution; the corresponding primary antibodies anti-NF-*κ*B p65 (ab16502, Abcam, Cambridge, 1 : 500), anti-p38 (Abcam, ab31828, 1 : 1000), anti-mTOR (phospho S2448)(Abcam, ab109268, 1 : 1000), phospho-PI3K p85 (AF3242, Affinity, 1 : 500), anti-AKT (phospho T308) (ab38449, Abcam, 1 : 500), anti-Bcl-2 (Affinity, AF6139, 1 : 1000), anti-Bax (Affinity, AF0120, 1 : 1000), anti-Cyt-C (Affinity, AF0146, 1 : 1000), anti-caspase-3 (Affinity, AF6311, 1 : 1000), anti-cleaved caspase-3 (Affinity, AF7022, 1 : 1000), and anti-GAPDH (ab8245, Abcam, 1 : 500) were added proportionally and incubated overnight at 4°C. After washing the membrane, a second antibody was added and incubated at room temperature for another 1 h. After washing the membrane again, the developer and fixative 1 : 1 were mixed; then, the protein strip was developed with a gel imaging system, and the statistical results were analyzed.

### 2.13. Statistical Analysis

Statistical analysis was performed using SPSS software (version 22.0). One-way ANOVA followed by SNK test was selected for multigroup difference analysis, while the Kruskal-Wallis *H* test was used when the variance was uneven. The data were expressed as mean ± SD (*P* < 0.05, statistically significant).

## 3. Results

### 3.1. Chemical Component Identification of FJD

**Figure 1:**
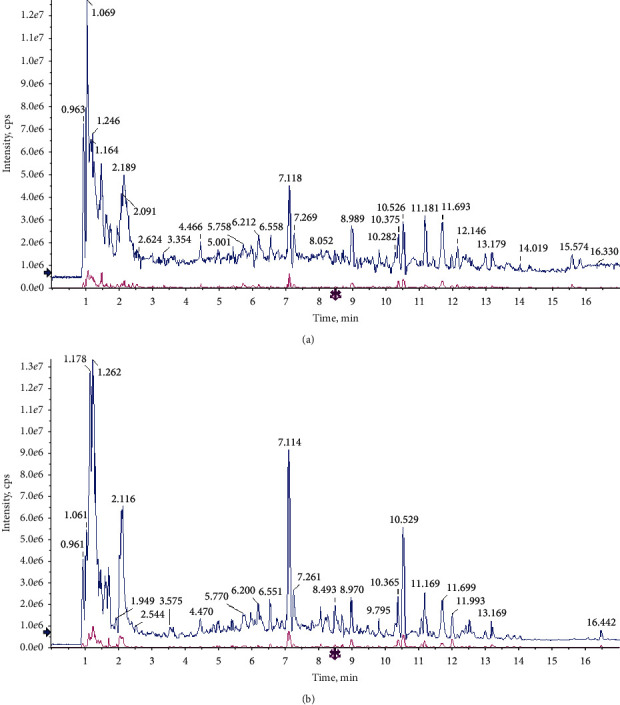
Chemical components of Fuzheng Jiedu decoction (FJD): (a) UPLC-Q/TOF-MS analysis in a positive mode; (b) UPLC-Q/TOF-MS analysis in a negative mode.

The chemical components of FJD were detected using the UPLC-Q/TOF MS method. As shown in Figure 1 and Table S1, the UPLC-Q/TOF MS analysis identified 68 compounds in a positive ion mode 61 compounds in a negative ion mode. These compounds in FJD mainly include acids, alkaloids, flavonoids, amino acids, polyphenols, etc.

### 3.2. Inhibitory Effect of FJD on Tumor Growth in SKOV3 Tumor-Bearing Mice

**Figure 2:**
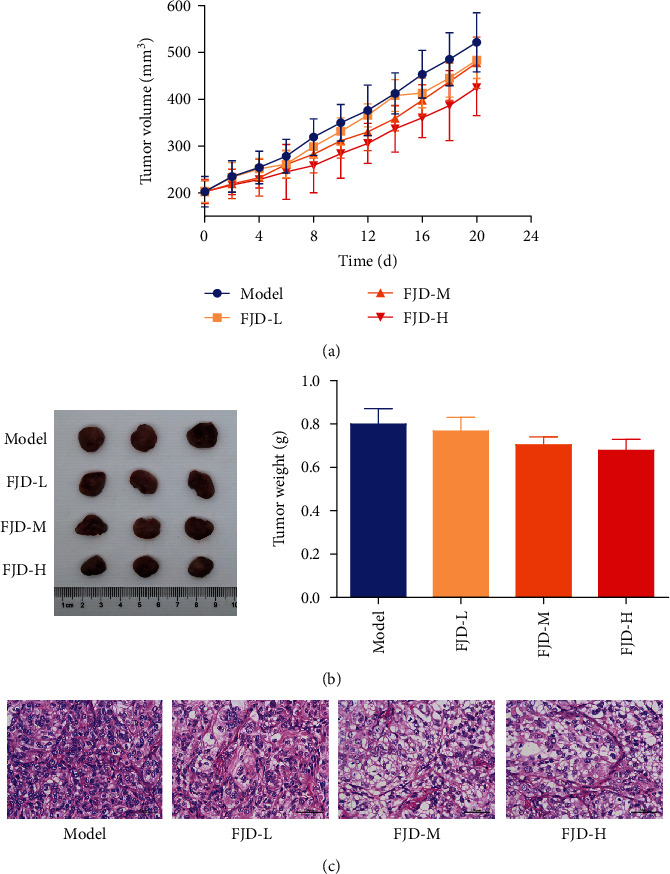
Effects of different doses of FJD on tumor volumes and weights and histopathology in OC tumor-bearing mice: (a) tumor volumes; (b) tumor weights; (c) HE staining of tumor tissues. Data was expressed as mean ± SD.

As shown in Figures 2(a) and 2(b), the tumor volumes in the model group gradually increased with time, while the treatments with different doses of FJD tend to reduce the tumor volumes and weights compared with the model group. However, there are no statistical differences for the tumor volumes and weights between the model and FJD groups. At the same time, we also took tumor tissues for pathological analysis. The results showed that compared with the model group, different doses of FJD aggravated the pathological damage of tumors to varying degrees, including the decrease in tumor cell numbers, partial tissue necrosis, and severe lymphocyte infiltration (Figure 2(c)).

### 3.3. Effects of FJD on the Bcl-2 Family and the PI3K/AKT/mTOR/NF-*κ*B Signaling Pathway in Tumor Tissues

**Figure 3:**
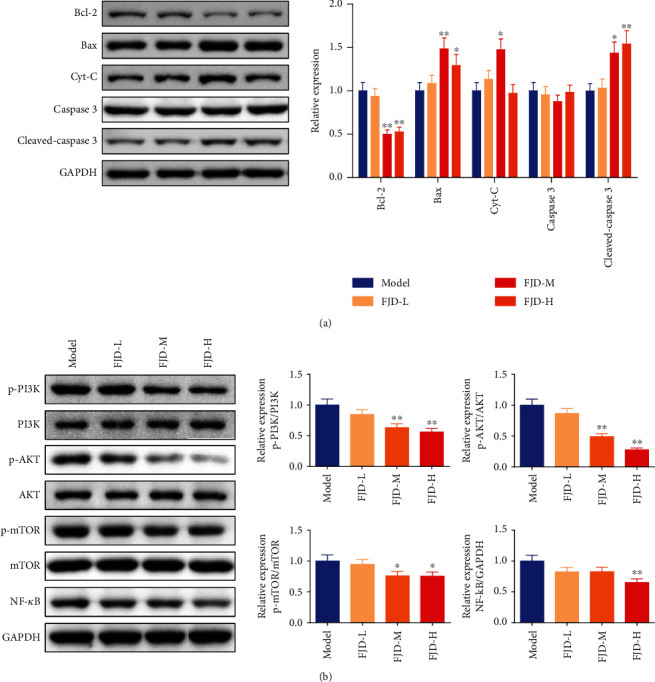
Effects of different doses of FJD on the Bcl-2 family protein (a) and the PI3K/AKT/mTOR/NF-*κ*B (b) signaling pathway of protein levels in tumor tissues. Data was expressed as mean ± SD. Compared with the model group, ^★^*P* < 0.05, ^★★^*P* < 0.01.

After detecting the inhibitory effect of FJD on tumor tissues, we further detected the changes of proteins related to the Bcl-2 family and the PI3K/AKT/mTOR/NF-*κ*B signaling pathway in tumor tissues. As shown in Figure 3(a), the protein expression of Bax, Cyt-C, and cleaved caspase-3 in the FJD-H group was evidently higher than that in the model group, while Bcl-2 was downregulated. Furthermore, the levels of p-PI3K/PI3K, p-AKT/AKT, and p-mTOR/mTOR in the FJD-M and FJD-H groups were obviously decreased compared with the model group, and the level of NF-*κ*B in the FJD-H group was significantly lowered (Figure 3(b)).

### 3.4. Inhibitory Effect of FJD Combined with Cisplatin on Tumor Growth in Tumor-Bearing Mice

**Figure 4:**
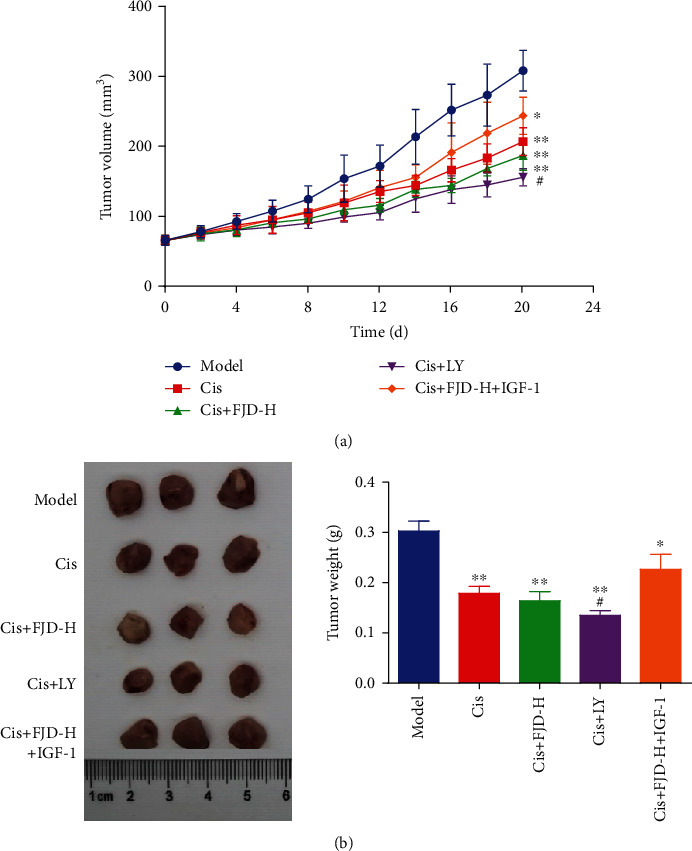
Cisplatin inhibited tumor volumes and weights and FJD enhancement effect in mice: (a) tumor volumes and (b) tumor weights. Data was expressed as mean ± SD. Compared with the model group, ^★^*P* < 0.05, ^★★^*P* < 0.01; compared with the Cis group, ^#^*P* < 0.05.

As shown in Figure 4(a), after 20 d of Cis treatment, the tumor volumes of SKOV3 cells decreased dramatically. FJD-H combined with Cis showed a trend of increasing the efficacy of Cis, and the tumor volumes were also lower than those of the model group. After giving the PI3K inhibitor LY294002 (LY), the effect of Cis+LY on reducing the tumor volume was stronger than that of Cis alone, indicating that PI3K may be involved in the process of OC tumor formation. Similarly, we also detected the PI3K activator IGF-1, although the tumor volume in the Cis+FJD-H+IGF-1 group mice was still higher than that in the model group, but it also hindered the tumor inhibitory effect of the Cis+FJD-H group as expected. Correspondingly, the tumor weights of Cis-participated groups were dramatically lower than those of the model group; FJD addition enhanced the antitumor effect of Cis, the same as LY, while IGF-1 partially reversed this antitumor effect (Figure 4(b)).

### 3.5. Regulation of the Bcl-2 Family Proteins and the PI3K/AKT/mTOR/NF-*κ*B Signaling Pathway in FJD Combined with Cisplatin in Tumor-Bearing Mice

**Figure 5:**
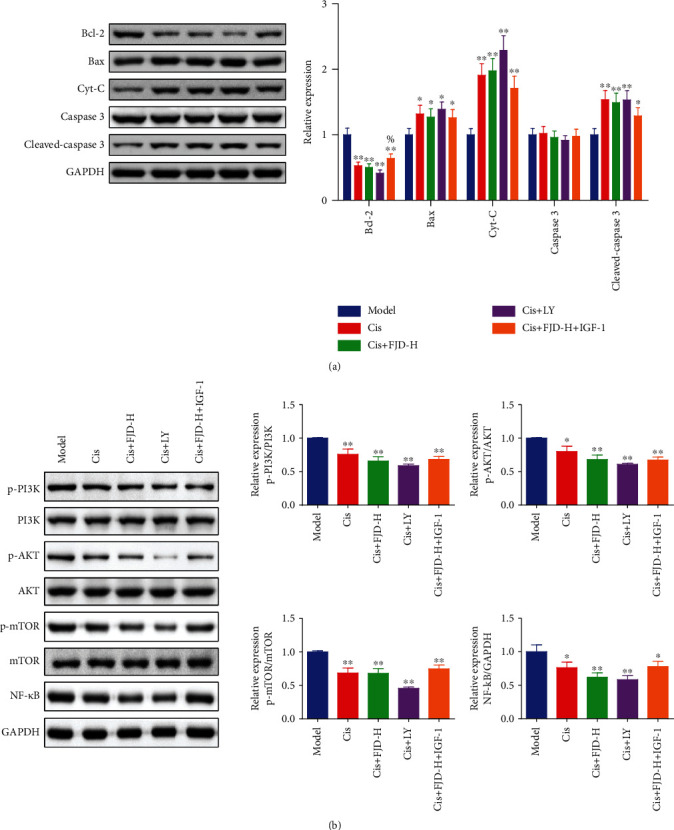
Regulatory effect of FJD combined with cisplatin on apoptosis-related proteins (a) and the PI3K/AKT/mTOR/NF-*κ*B (b) signaling pathway in the animal model. Data was expressed as mean ± SD. Compared with the model group, ^★^*P* < 0.05, ^★★^*P* < 0.01; compared with the Cis+FJD-H group, ^%^*P* < 0.05.

As shown in Figure 5(a), the Bcl-2 family proteins responded positively regardless of cisplatin alone or in combination with other drugs. Specifically, the protein expression of the proapoptotic proteins (Bax, Cyt-C, and cleaved caspase-3) was upregulated, and the antiapoptotic protein (Bcl-2) was downregulated, indicating that cisplatin could promote the tumor cell apoptosis. In Figure 5(b), the protein levels of the p-PI3K/PI3K, p-AKT/AKT, p-mTOR/mTOR, and NF-*κ*B were decreased significantly after Cis treatment compared with the model group; this effect was further enhanced by LY or FJD. However, in the Cis+FJD-H+IGF-1 group, the addition of IGF-1 recovered the decreased levels of these proteins compared with the Cis+FJD-H group.

### 3.6. FJD Inhibited the Growth, Invasion, and Apoptosis of OC Cell Lines SKOV3 and 3AO

**Figure 6:**
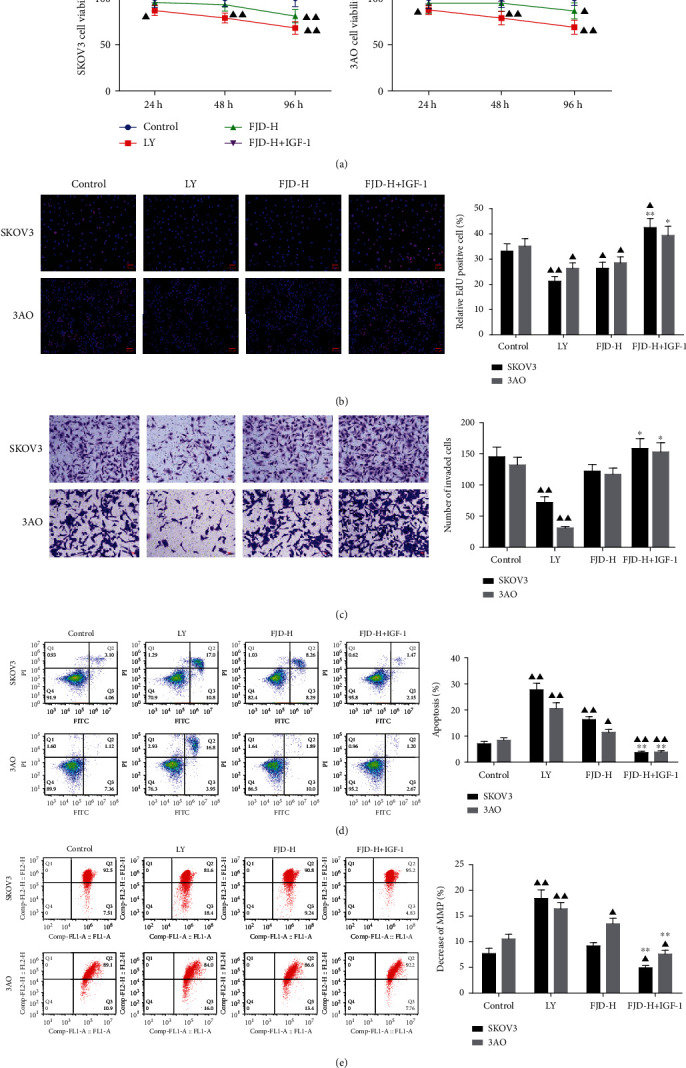
FJD inhibited the cell proliferation, invasion, and apoptosis of two OC cell lines SKOV-3 and 3AO: (a) time variation in viability of SKOV-3 and 3AO cells; (b) EdU assessed cell proliferation toxicity; (c) changes of invasion ability of SKOV3 and 3AO cells in each group (200x); (d) the cell apoptosis was detected by the Annexin V-FITC/PI staining with flow cytometry; (e) the mitochondrial transmembrane potential was detected by the JC-1 staining with flow cytometry. Data was expressed as mean ± SD. Compared with the control group, ^▲^*P* < 0.05, ^▲▲^*P* < 0.01; compared with the FJD-H group, ^∗^*P* < 0.05, ^∗∗^*P* < 0.01).

According to the results of animal experiments, we also made some validation at the cellular level. As shown in Figure 6(a), in SKOV3 and 3AO cells, the cell viability of the LY group at any time (24, 48, and 96 h) was evidently below than that of the control group. For the FJD-H group, the cell viability was decreased obviously at 96 h, while the cell viability was increased significantly in the FJD-H+IGF-1 group, which exceeded the control group. The cell proliferation evaluation using the EdU assay was consistent with the above results. In Figure 6(b), we observed that FJD-H and LY have similar inhibitory effects on the SKOV3 and 3AO cell proliferation, while IGF-1 had opposite effect. Similarly, compared with the control group, the OC cell invasion ability of the LY group was significantly reduced, while FJD-H had no significant effect on cell invasion. Compared with the FJD-H group, the FJD-H+IGF-1 group increased the invasion numbers of the SKOV3 and 3AO cells (Figure 6(c)). In Figures 6(d) and 6(e), compared with the control group, the apoptosis of SKOV3 and 3AO cells was increased in the LY group and FJD-H group, while it was decreased significantly in the FJD-H+IGF-1 group. Moreover, the mitochondrial membrane potential (MMP) is also an indicator of apoptosis; the results of MMP in different groups in Figure 6(e) were consistent with the tendencies in Figure 6(c).

### 3.7. FJD Promoted the OC Cell Apoptosis by Regulating the PI3K/AKT/mTOR/P38 Signaling Pathway

**Figure 7:**
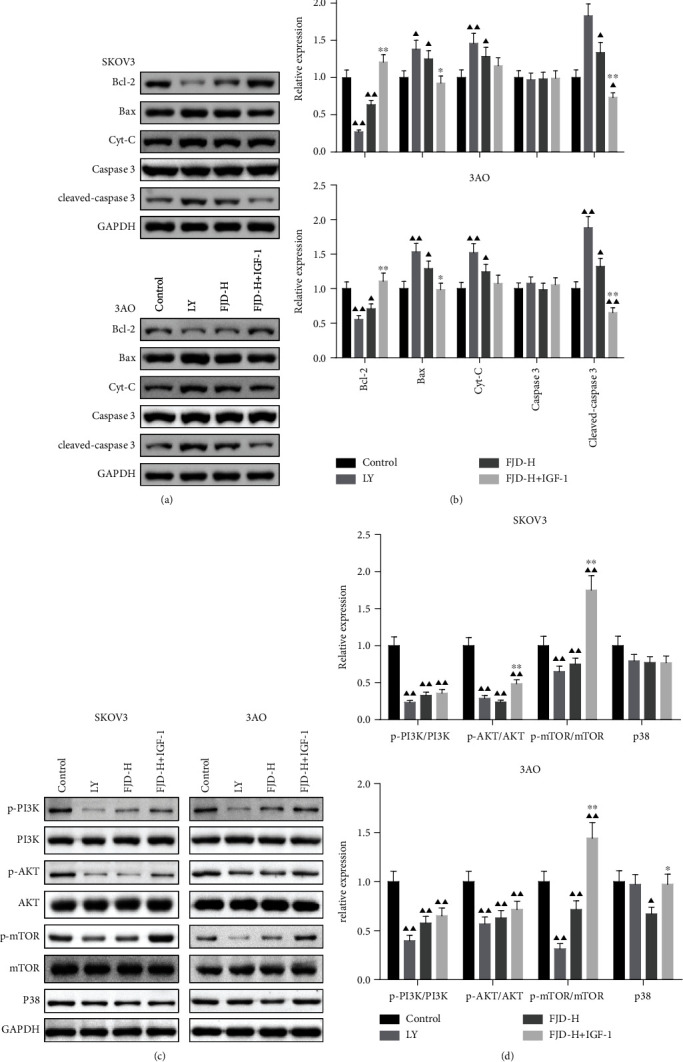
FJD p6romoted OC cell apoptosis by regulating the PI3K/AKT/mTOR/P38 signaling pathway: (a, b) the expressions of Bax, Cyt-C, caspase-3, cleaved caspase-3, and Bcl-2 in OC cells; (c, d) the protein levels of p-PI3K/PI3K, p-AKT/AKT, p-mTOR/mTOR, and p38 in SKOV3 and 3AO cells, respectively. Data was expressed as mean ± SD. Compared with the control group, ^▲^*P* < 0.05, ^▲▲^*P* < 0.01; compared with the FJD-H group, ^∗^*P* < 0.05, ^∗∗^*P* < 0.01.

In accordance with the results of apoptotic protein in tumor tissues, FJD treatment increased the levels of Bax, Cyt-C, and cleaved caspase-3 and reduced Bcl-2 in SKOV3 and 3AO cells (Figures 7(a) and 7(b)). Besides, the protein levels of p-PI3K/PI3K, p-AKT/AKT, and p-mTOR/mTOR in LY and FJD-H groups were reduced significantly compared with the control group. However, the levels of p-AKT/AKT and p-mTOR/mTOR in the FJD-H+IGF-1 group were obviously higher than those in the FJD-H group in OC cells of SKOV3 and/or 3AO. Although the p38 level did not change at any treatment groups in SKOV3 cell lines, we observed that the p38 level in the FJD-H group was significantly lower than that in the control group in 3AO cell lines (Figures 7(c) and 7(d)).

### 3.8. FJD Enhanced the Inhibitory Effect of Cisplatin on the Cell Proliferation, Invasion, and Apoptosis of OC Cells

**Figure 8:**
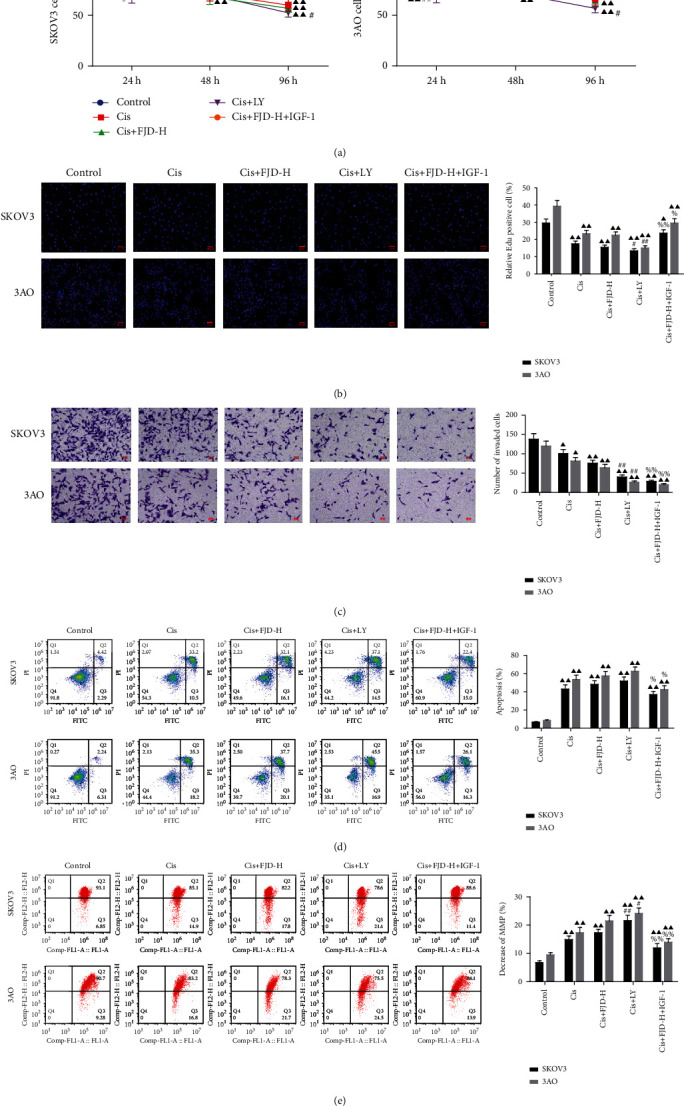
Inhibitory effect of cisplatin on the cell growth, invasion, and apoptosis of SKOV-3 and 3AO cells and FJD enhancing the efficacy of cisplatin: (a) the cell viability of SKOV-3 and 3AO cells; (b) EdU assessed cell proliferation toxicity; (c) the changes of cell invasion ability; (d) the cell apoptosis was detected by the Annexin V-FITC/PI staining with flow cytometry; (e) the mitochondrial transmembrane potential was detected by the JC-1 staining with flow cytometry. Data was expressed as mean ± SD. Compared with the control group, ^▲^*P* < 0.05, ^▲▲^*P* < 0.01; compared with the Cis group, ^#^*P* < 0.05, ^##^*P* < 0.05; compared with the Cis+FJD-H group, ^%^*P* < 0.05, ^%%^*P* < 0.01.

As shown in Figure 8(a), the cell viability of OC cell lines SKOV3 and 3AO in each treatment group was dramatically decreased compared with the control group at any time point, and the degree of reduction was positively correlated with the time of coincubation. At 24 or 96 h, LY reduced the cell viability of two OC cell lines by promoting the therapeutic effect of Cis in the Cis+LY group, while compared with the Cis+FJD-H group, the cell viability in the Cis+FJD-H+IGF-1 group was increased at 96 h. In Figure 8(b), combined treatment with cisplatin and FJD or LY significantly inhibited the proliferation of OC cells, and this effect was weakened by IGF-1. The cell invasion results were similar to those mentioned above. Compared with the control group, invaded cell numbers in the Cis, Cis+FHD-H, and Cis+LY groups were significantly decreased, while compared with the Cis+FJD-H group, the Cis+FJD-H+IGF-1 group reduced the number of invaded cells (Figure 8(c)). The apoptosis of SKOV3 and 3AO cells in each group was significantly higher than that in the control group, and the Cis+FJD-H+IGF-1 group was lower than the Cis+FJD-H group (Figure 8(d)). By evaluating the MMP, we confirmed that cisplatin in combination with FJD or LY enhanced the effect of cisplatin and dramatically reduced the MMP in two OC cell lines. On the contrary, IGF-1 partly reversed the efficacy of FJD-H and cisplatin in reducing MMP (Figure 8(e)).

### 3.9. FJD Enhanced the Proapoptotic Effect of Cisplatin by the PI3K/AKT/mTOR Signaling Pathway

**Figure 9:**
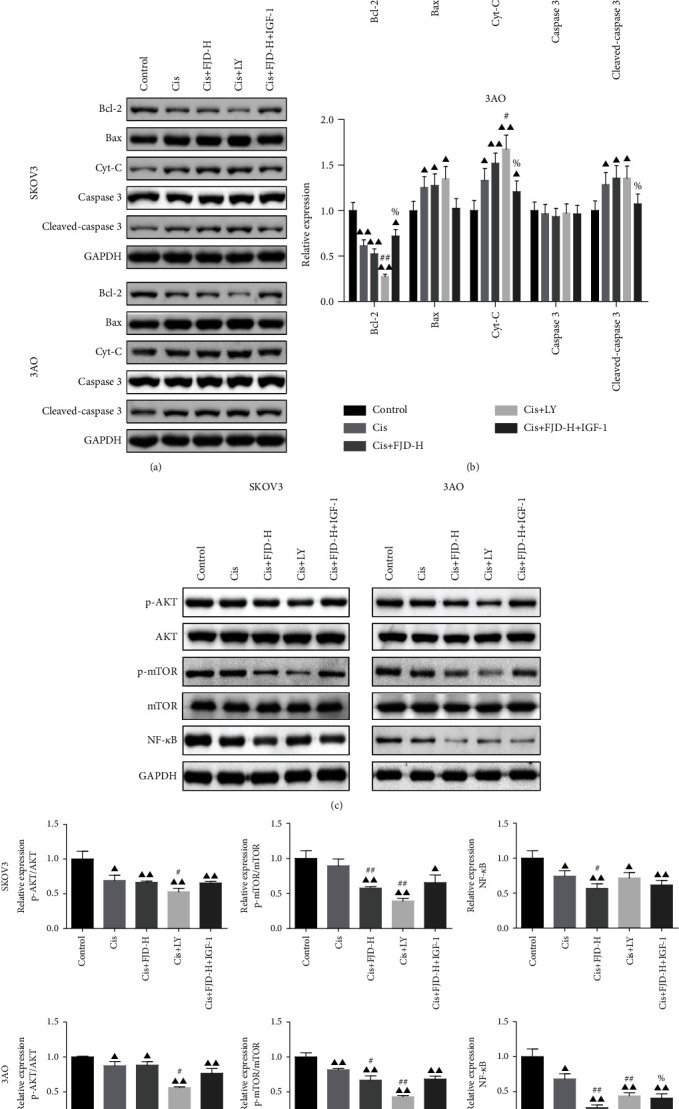
Effect of FJD combined with cisplatin on the apoptosis and the PI3K/AKT/mTOR signaling pathway at the cell level: (a, b) the expressions of Bax, Cyt-C, caspase-3, cleaved caspase-3, and Bcl-2 in two OC cell lines; (c, d) the protein levels of p-PI3K/PI3K, p-AKT/AKT, and p-mTOR/mTOR in SKOV3 and 3AO cells were detected, respectively. Data was expressed as mean ± SD. Compared with the control group, ^▲^*P* < 0.05, ^▲▲^*P* < 0.01; compared with the Cis group, ^#^*P* < 0.05, ^##^*P* < 0.05; compared with the Cis+FJD-H group, ^%^*P* < 0.05.

We also detected the effect of FJD and cisplatin on OC cell apoptosis. As shown in Figures 9(a) and 9(b), cisplatin and FJD cotreatment promoted the expressions of proapoptotic proteins; the expression of Cyt-C in the Cis+FJD-H group was higher than that in Cis alone group. In addition, compared with the Cis+FJD-H group, IGF-1 reversed the expression tendency of Cyt-C, cleaved caspase-3, and Bcl-2. In addition, at each treatment group in two OC cell lines, the protein levels of p-PI3K/PI3K, p-AKT/AKT, and p-mTOR/mTOR were decreased in varying degrees. Among these groups, the levels of most of these proteins were further decreased in the Cis+LY group and Cis+FJD-H group compared with the Cis alone. IGF-1 addition recovered the decreased expression of the partial of these proteins compared with the Cis+FJD-H (Figures 9(c) and 9(d)).

## 4. Discussion

OC, one of the deadliest gynecological cancers, is the seventh most common cancer in women worldwide and ranks eighth in overall cancers, with more than 239,000 new cases each year [22]. Like other general tumors, unlimited proliferation is the main reason for the high mortality of OC. Therefore, the research and development of antiovarian cancer drugs are an urgent problem to be solved. FJD is valuable and widely used as a TCM formula for the treatment of tumors [17–20]. In this study, we focused on the anticancer activities of FJD alone and in combination with cisplatin in OC cells *in vivo* and *in vitro*, as well as its potential in regulating the PI3K/AKT/mTOR/NF-*κ*B signaling pathway. We confirmed that FJD could improve the antitumor efficacy of cisplatin by inhibiting the PI3K/AKT/mTOR/NF-*κ*B signal pathway. This study may help to provide a novel idea and a theoretical basis for the treatment of OC by TCM.

TCM or compounds from TCM have been reported to be of value in the treatment of OC and have achieved good effects [23, 24]. Liu et al. reported that dihydroartemisinin, a derivative of artemisinin that was separated from the TCM herb *Artemisia annua* L., could inhibit the proliferation, migration, and invasion of epithelial OC [25]. Tao et al. found that the application of TCM *Poria cocos* inhibited the proliferation of OC cells, tumor growth, and metastasis *in vivo* [26]. In our research, although FJD had no significant inhibitory effect on OC tumor growth, the results confirmed that FJD could inhibit the cell proliferation, promote apoptosis, and enhance the anticancer effect of cisplatin on OC *in vivo* and *in vitro*. This indicates that FJD has the opportunity to be used as an adjunct in clinical treatment of cancer to enhance the efficacy of chemotherapy drugs.

Many previous studies have reported that the PI3K/AKT/mTOR/NF-*κ*B pathway is closely related to the occurrence of OC [8, 27, 28]. In carboplatin-induced ovarian cancer cells, CYR61 regulates the protein levels of p53 and NF-*κ*B through the PI3K/AKT/mTOR pathway [29]. Icariin inhibited the proliferation, migration, and invasion of OC cells SKOV3 *in vitro* and promoted the OC cell apoptosis by inhibiting the PI3K/AKT signaling pathway [23]. Therefore, we also hypothesized that FJD's inhibition effect on OC was related to the PI3K/AKT/mTOR/NF-*κ*B signaling pathway. Our research also confirmed the conjecture that FJD significantly reduced the protein levels of p-PI3K/PI3K, p-AKT/AKT, p-mTOR/mTOR, NF-*κ*B, and p38 *in vivo* and *in vitro* of OC. In addition, the cotreatment with the PI3K inhibitor LY or activator IGF-1 also confirmed the involvement of the PI3K/AKT/mTOR/NF-*κ*B signaling pathway in inhibiting the OC by FJD.

Cisplatin is widely used as a clinical anticancer drug and has been reported as one of the most effective chemotherapeutic drugs for OC [30, 31]. Choi et al. confirmed that *Scutellaria baicalensis* combined with cisplatin has a synergistic anticancer effect on human OC cells [32]. Kan et al. reported that sulforaphane combined with cisplatin could promote the apoptosis of OC cells by upregulating Bcl-2 expression and downregulating Bax and cleaved caspase-3 [33]. However, the resistance of conventional chemotherapeutic drugs also limits the use of cisplatin [34–36]. This study found that cisplatin inhibited the OC tumor formation, increased the expressions of proapoptotic proteins Bax, Cyt-C, and cleaved caspase-3, and downregulated the level of the antiapoptotic protein Bcl-2. And with the simultaneous administration of FJD and cisplatin, the antitumor efficacy of cisplatin was enhanced, and the levels of PI3K/AKT/mTOR/NF-*κ*B signaling-related proteins were further suppressed. It indicates that FJD could relieve the cisplatin resistance to enhance the anticancer effect of cisplatin to a certain extent [37, 38].

In summary, based on the *in vivo* and *in vitro* experiments, our study showed that FJD improved the anticancer effect of cisplatin on OC by promoting the cell apoptosis and regulating the PI3K/AKT/mTOR/NF-*κ*B signal pathway. Thus, it is suggested that FJD may be used as an adjuvant or targeted drug in the treatment of OC. However, this study also has limitations. In *in vivo* study, we only established the subcutaneous tumor xenograft model to investigate the antitumor effect of FJD; the role of FJD in orthotopic transplantation of the OC tumor needs to be investigated in our further study. In addition, because of the diverse etiology and complex pathogenesis of OC, the clinical value of FJD remains to be further studied and confirmed.

## 5. Conclusion

This study confirmed that FJD administration could inhibit the proliferation of OC cells and promote cell apoptosis. Moreover, FJD combined with cisplatin have a synergistic anticancer effect on OC, and this enhancement effect is achieved by inhibiting the PI3K/AKT/mTOR/NF-*κ*B signal pathway *in vivo* and *in vitro*. Our findings may provide a theoretical basis for further research on the pathogenesis and treatment of OC.

## Data Availability

All data are included in the article.

## Supplementary Materials

Supplementary Materials Table S1: chemical components of FJD using the UPLC-Q/TOF-MS method.
