# Supplementary material for: Fuzheng Jiedu Decoction Induces Apoptosis and Enhances Cisplatin Efficacy in Ovarian Cancer Cells In Vitro and In Vivo through Inhibiting the PI3K/AKT/mTOR/NF-κB Signaling Pathway
Source: Biomed Res Int. 2022 Mar 2;2022:5739909. doi: 10.1155/2022/5739909 (PMC8906977; doi:10.1155/2022/5739909)
Supplement: Supplementary Materials — Table S1: chemical components of FJD using the UPLC-Q/TOF-MS method. [file 5739909.f1.docx]

**Table S1**. Chemical components of FJD using the UPLC-Q/TOF-MS method

|  | Component Name | Area | Retention Time | Formula | Precursor Mass | Found At Mass | Mass Error (ppm) | Library Score | Isotope Ratio Difference |
| --- | --- | --- | --- | --- | --- | --- | --- | --- | --- |
| Positive iron mode | | | | | | | | | |
| 1 | Histidine | 102000 | 1.08 | C_6_H_9_N_3_O_2_ | 156.077 | 156.0767 | 0.0 | 95.7 | 2.5 |
| 2 | Glutamic acid | 426300 | 1.15 | C_5_H_9_NO_4_ | 148.060 | 148.0605 | 0.1 | 95.4 | 1.8 |
| 3 | L-Valine | 2112000 | 1.16 | C_5_H_11_NO_2_ | 118.086 | 118.0861 | -1.0 | 100.0 | 0.3 |
| 4 | Trigonelline | 939700 | 1.21 | C_7_H_7_NO_2_ | 138.055 | 138.0549 | -0.4 | 93.8 | 0.8 |
| 5 | Proline | 2731000 | 1.23 | C_5_H_9_NO_2_ | 116.071 | 116.0704 | -1.3 | 96.9 | 0.6 |
| 6 | Stachydrine | 485000 | 1.28 | C_7_H_13_NO_2_ | 144.102 | 144.1018 | -0.5 | 97.2 | 0.5 |
| 7 | Adenine | 1166000 | 1.49 | C_5_H_5_N_5_ | 136.062 | 136.0615 | -1.7 | 97.3 | 1.2 |
| 8 | Cytidine | 181100 | 1.56 | C_9_H_13_N_3_O_5_ | 244.093 | 244.0930 | 0.6 | 100.0 | 3.0 |
| 9 | Nicotinic acid | 280700 | 1.70 | C_6_H_5_NO_2_ | 124.039 | 124.0392 | -0.9 | 95.5 | 0.7 |
| 10 | Nicotinamide | 190400 | 1.81 | C_6_H_6_N_2_O | 123.055 | 123.0552 | -0.8 | 91.7 | 1.1 |
| 11 | 6-Hydroxypurine | 130100 | 1.88 | C_5_H_4_N_4_O | 137.046 | 137.0457 | -1.0 | 98.8 | 2.1 |
| 12 | Adenosine | 718800 | 2.43 | C_10_H_13_N_5_O_4_ | 268.104 | 268.1040 | -0.1 | 100.0 | 0.1 |
| 13 | Guanosine | 219800 | 2.55 | C_10_H_13_N_5_O_5_ | 284.099 | 284.0991 | 0.4 | 100.0 | 1.2 |
| 14 | Lithospermoside | 11090 | 2.87 | C_14_H_19_NO_8_ | 330.118 | 330.1188 | 1.4 | 73.2 | 4.1 |
| 15 | Matrine | 34880 | 3.28 | C_15_H_24_N_2_O | 249.196 | 249.1963 | 0.4 | 97.2 | 0.3 |
| 16 | Phenylalanine | 373400 | 3.36 | C_9_H_11_NO_2_ | 166.086 | 166.0861 | -0.9 | 98.9 | 0.3 |
| 17 | Harpagide +NH3 | 36240 | 3.60 | C_15_H_24_O_10_.NH_3_ | 382.171 | 382.1708 | 0.2 | 89.8 | 3.1 |
| 18 | DL-Ephedrine | 59940 | 4.51 | C_10_H_15_NO | 166.123 | 166.1226 | -0.1 | 98.1 | 1.3 |
| 19 | Vitexin | 73180 | 4.84 | C_21_H_20_O_10_ | 433.113 | 433.1133 | 0.9 | 96.6 | 1.3 |
| 20 | Chlorogenic acid | 195600 | 4.98 | C_16_H_18_O_9_ | 355.102 | 355.1024 | 0.1 | 100.0 | 1.4 |
| 21 | Epicatechin | 362300 | 5.03 | C_15_H_14_O_6_ | 291.086 | 291.0860 | -0.9 | 96.2 | 0.9 |
| 22 | Procyanidin B2 | 605600 | 5.39 | C_30_H_26_O_12_ | 579.150 | 579.1498 | 0.1 | 95.9 | 1.0 |
| 23 | Fraxetin | 886000 | 5.44 | C_10_H_8_O_5_ | 209.044 | 209.0443 | -0.6 | 93.7 | 0.3 |
| 24 | Puerarin | 657600 | 5.53 | C_21_H_20_O_9_ | 417.118 | 417.1182 | 0.4 | 96.5 | 0.1 |
| 25 | Hamaudol Glycoside | 51470 | 5.63 | C_21_H_26_O_10_ | 439.160 | 439.1603 | 1.0 | 84.7 | 1.9 |
| 26 | Norisoboldine | 666900 | 5.73 | C_18_H_19_NO_4_ | 314.139 | 314.1385 | -0.7 | 95.6 | 0.3 |
| 27 | Sweroside | 217100 | 5.99 | C_16_H_22_O_9_ | 359.134 | 359.1339 | 0.8 | 97.6 | 1.3 |
| 28 | Albiflorin | 680000 | 6.21 | C_23_H_28_O_11_ | 481.170 | 481.1705 | 0.0 | 93.8 | 2.8 |
| 29 | Schaftoside | 902400 | 6.27 | C_26_H_28_O_14_ | 565.155 | 565.1555 | 0.5 | 92.6 | 3.7 |
| 30 | Prim-O-glucosylcimifugin | 23580 | 6.49 | C_22_H_28_O_11_ | 469.170 | 469.1708 | 0.9 | 86.8 | 3.5 |
| 31 | 6-Methylcoumarin | 51910 | 6.56 | C_10_H_8_O_2_ | 161.060 | 161.0596 | -0.8 | 72.3 | 3.1 |
| 32 | Paeoniflorin +NH3 | 431400 | 6.56 | C_23_H_28_O_11_.NH_3_ | 498.197 | 498.1973 | 0.6 | 93.5 | 3.5 |
| 33 | Resveratrol | 7249000 | 7.12 | C_14_H_12_O_3_ | 229.086 | 229.0858 | -0.6 | 91.1 | 0.5 |
| 34 | Polydatin | 975000 | 7.12 | C_20_H_22_O_8_ | 391.139 | 391.1386 | -0.4 | 92.7 | 0.4 |
| 35 | (-)-Catechin Gallate | 619600 | 7.26 | C_22_H_18_O_10_ | 443.097 | 443.0975 | 0.5 | 96.9 | 0.5 |
| 36 | Liquiritigenin | 224300 | 7.26 | C_15_H_12_O_4_ | 257.081 | 257.0810 | 0.5 | 89.5 | 0.1 |
| 37 | Scopoletin | 173700 | 7.27 | C_10_H_8_O_4_ | 193.050 | 193.0495 | 0.0 | 94.9 | 0.3 |
| 38 | Dihydroquercetin | 58700 | 7.50 | C_15_H_12_O_7_ | 305.066 | 305.0660 | 1.2 | 92.0 | 0.5 |
| 39 | 4'-O-β-Glucopyranosyl-5-O-methylvisamminol | 90690 | 7.93 | C_22_H_28_O_10_ | 453.176 | 453.1759 | 0.9 | 97.0 | 1.6 |
| 40 | Narirutin | 33000 | 8.06 | C_27_H_32_O_14_ | 581.186 | 581.1874 | 1.6 | 87.4 | 1.4 |
| 41 | Hesperetin | 76810 | 8.22 | C_16_H_14_O_6_ | 303.086 | 303.0865 | 0.7 | 94.1 | 1.4 |
| 42 | Hesperidin | 41250 | 8.55 | C_28_H_34_O_15_ | 611.197 | 611.1975 | 0.7 | 79.5 | 0.4 |
| 43 | Peiminine | 9616 | 8.72 | C_27_H_43_NO_3_ | 430.332 | 430.3313 | -0.7 | 100.0 | 2.1 |
| 44 | Tracheloside +NH3 | 123500 | 8.81 | C_27_H_34_O_12_.NH_3_ | 568.239 | 568.2397 | 1.4 | 95.6 | 2.5 |
| 45 | Xanthotoxol | 17310 | 8.97 | C_11_H_6_O_4_ | 203.034 | 203.0342 | 1.6 | 81.1 | 3.2 |
| 46 | Scutellarin | 381800 | 9.01 | C_21_H_18_O_12_ | 463.087 | 463.0877 | 1.3 | 100.0 | 1.6 |
| 47 | Benzoylmesaconine | 1734000 | 9.02 | C_31_H_43_NO_10_ | 590.296 | 590.2962 | 0.5 | 98.6 | 2.8 |
| 48 | Scutellarein | 79010 | 9.03 | C_15_H_10_O_6_ | 287.055 | 287.0549 | -0.4 | 98.9 | 2.5 |
| 49 | Baicalin | 430000 | 9.26 | C_21_H_18_O_11_ | 447.092 | 447.0925 | 0.7 | 100.0 | 1.2 |
| 50 | Daidzein | 200300 | 9.54 | C_15_H_10_O_4_ | 255.065 | 255.0650 | -0.5 | 97.2 | 0.3 |
| 51 | Berberine | 107900 | 9.93 | C_20_H_17_NO_4_ | 336.123 | 336.1229 | -0.3 | 93.2 | 0.4 |
| 52 | Benzoylhypacoitine | 615700 | 10.04 | C_31_H_43_NO_9_ | 574.301 | 574.3014 | 0.6 | 100.0 | 1.8 |
| 53 | Oxypeucedanin hydrate | 95900 | 10.06 | C_16_H_16_O_6_ | 305.102 | 305.1023 | 0.9 | 82.3 | 0.6 |
| 54 | Nodakenin | 1023000 | 10.38 | C_20_H_24_O_9_ | 409.149 | 409.1498 | 1.1 | 91.0 | 0.8 |
| 55 | Emodin | 4539000 | 10.53 | C_15_H_10_O_5_ | 271.060 | 271.0599 | -0.9 | 99.7 | 1.2 |
| 56 | Wogonin 7-O-glucuronide | 120300 | 10.70 | C_22_H_20_O_11_ | 461.108 | 461.1082 | 0.7 | 100.0 | 1.0 |
| 57 | Wilforlide A | 715100 | 11.18 | C_30_H_46_O_3_ | 455.352 | 455.3519 | -0.3 | 82.6 | 2.1 |
| 58 | Angelicin | 149800 | 11.28 | C_11_H_6_O_3_ | 187.039 | 187.0389 | -0.2 | 98.6 | 0.4 |
| 59 | Paeonol | 193300 | 12.34 | C_9_H_10_O_3_ | 167.070 | 167.0701 | -0.8 | 89.0 | 1.2 |
| 60 | Formononetin | 283000 | 12.83 | C_16_H_12_O_4_ | 269.081 | 269.0806 | -0.9 | 92.7 | 0.8 |
| 61 | Dictamnine | 49050 | 12.84 | C_12_H_9_NO_2_ | 200.071 | 200.0706 | -0.1 | 91.9 | 1.1 |
| 62 | Glycyrrhizic acid | 500500 | 13.18 | C_42_H_62_O_16_ | 823.411 | 823.4118 | 0.9 | 95.2 | 1.2 |
| 63 | Nobiletin | 370400 | 14.30 | C_21_H_22_O_8_ | 403.139 | 403.1390 | 0.5 | 79.2 | 1.8 |
| 64 | Neobavaisoflavone | 32840 | 14.99 | C_20_H_18_O_4_ | 323.128 | 323.1280 | 0.7 | 97.6 | 1.1 |
| 65 | A 3-N-butyl-4,5-dihydrophthalide | 86970 | 15.44 | C_12_H_16_O_2_ | 193.122 | 193.1222 | -0.5 | 93.2 | 1.5 |
| 66 | Tangeretin | 141700 | 15.46 | C_20_H_20_O_7_ | 373.128 | 373.1283 | 0.4 | 86.2 | 0.3 |
| 67 | Ligustilide | 110600 | 16.59 | C_12_H_14_O_2_ | 191.107 | 191.1066 | -0.4 | 79.7 | 1.3 |
| 68 | Isoalantolactone | 171100 | 16.90 | C_15_H_20_O_2_ | 233.154 | 233.1536 | -0.1 | 93.6 | 1.9 |
| Negative iron mode | | | | | | | | | |
| 1 | L(+)-Arginine | 471600 | 1.06 | C_6_H_14_N_4_O_2_ | 173.104 | 173.1046 | 1.0 | 99.2 | 0.2 |
| 2 | Histidine | 43460 | 1.06 | C_6_H_9_N_3_O_2_ | 154.062 | 154.0622 | -0.3 | 74.3 | 1.6 |
| 3 | Quinic acid | 1266000 | 1.23 | C_7_H_12_O_6_ | 191.056 | 191.0559 | -1.2 | 87.6 | 0.6 |
| 4 | L-Malic acid | 1035000 | 1.43 | C_4_H_6_O_5_ | 133.014 | 133.0142 | -0.6 | 74.5 | 0.6 |
| 5 | Citric acid | 2954000 | 1.96 | C_6_H_8_O_7_ | 191.020 | 191.0197 | 0.1 | 98.2 | 0.3 |
| 6 | Amber Acid | 93570 | 2.33 | C_4_H_6_O_4_ | 117.019 | 117.0192 | -1.2 | 93.1 | 0.1 |
| 7 | Guanosine | 242200 | 2.55 | C_10_H_13_N_5_O_5_ | 282.084 | 282.0845 | 0.3 | 99.3 | 0.0 |
| 8 | Leucine | 44710 | 2.56 | C_6_H_13_NO_2_ | 130.087 | 130.0873 | -0.7 | 100.0 | 0.2 |
| 9 | Gallic acid | 517500 | 2.71 | C_7_H_6_O_5_ | 169.014 | 169.0143 | 0.0 | 79.2 | 0.4 |
| 10 | Phenylalanine | 90360 | 3.36 | C_9_H_11_NO_2_ | 164.072 | 164.0715 | -1.0 | 98.5 | 0.4 |
| 11 | Danshensu | 50580 | 3.62 | C_9_H_10_O_5_ | 197.046 | 197.0454 | -0.9 | 87.3 | 0.2 |
| 12 | Protocatechuic acid | 75170 | 3.90 | C_7_H_6_O_4_ | 153.019 | 153.0191 | -1.2 | 93.4 | 0.4 |
| 13 | Esculin hydrate | 23500 | 4.46 | C_15_H_16_O_9_ | 339.072 | 339.0721 | -0.1 | 91.2 | 2.4 |
| 14 | L-Tryptophan | 160100 | 4.47 | C_11_H_12_N_2_O_2_ | 203.083 | 203.0825 | -0.5 | 96.2 | 1.6 |
| 15 | 8-Epiloganic acid | 1003000 | 4.49 | C_16_H_24_O_10_ | 375.130 | 375.1298 | 0.4 | 95.2 | 0.4 |
| 16 | Vitamin B2 | 1014000 | 4.49 | C_17_H_20_N_4_O_6_ | 375.131 | 375.1298 | -3.2 | 95.2 | 2.6 |
| 17 | Protocatechuic Aldehyde | 219200 | 4.89 | C_7_H_6_O_3_ | 137.024 | 137.0243 | -0.7 | 96.3 | 0.3 |
| 18 | Chlorogenic acid | 593600 | 4.98 | C_16_H_18_O_9_ | 353.088 | 353.0879 | 0.3 | 99.4 | 0.1 |
| 19 | Procyanidin B2 | 700400 | 5.39 | C_30_H_26_O_12_ | 577.135 | 577.1352 | 0.1 | 95.0 | 0.9 |
| 20 | Fraxin | 1373000 | 5.44 | C_16_H_18_O_10_ | 369.083 | 369.0830 | 0.8 | 97.8 | 0.2 |
| 21 | Esculetin | 30040 | 5.48 | C_9_H_6_O_4_ | 177.019 | 177.0192 | -0.7 | 74.4 | 0.2 |
| 22 | Puerarin | 453800 | 5.52 | C_21_H_20_O_9_ | 415.103 | 415.1036 | 0.4 | 99.5 | 0.1 |
| 23 | Caffeic acid | 117000 | 5.57 | C_9_H_8_O_4_ | 179.035 | 179.0348 | -0.8 | 78.9 | 0.4 |
| 24 | Catechin | 812700 | 5.81 | C_15_H_14_O_6_ | 289.072 | 289.0718 | 0.1 | 91.5 | 1.2 |
| 25 | Schaftoside | 541700 | 6.27 | C_26_H_28_O_14_ | 563.141 | 563.1405 | -0.2 | 93.7 | 0.6 |
| 26 | Daidzin +HCOOH | 65710 | 6.31 | C_21_H_20_O_9_.HCOOH | 461.109 | 461.1086 | -0.6 | 98.6 | 2.2 |
| 27 | Eleutheroside E +HCOOH | 10050 | 6.48 | C_34_H_46_O_18_.HCOOH | 787.267 | 787.2663 | -0.4 | 95.7 | 3.8 |
| 28 | Paeoniflorin +HCOOH | 2148000 | 6.56 | C_23_H_28_O_11_.HCOOH | 525.161 | 525.1613 | 0.0 | 99.0 | 1.6 |
| 29 | p-Coumaric acid | 77830 | 6.77 | C_9_H_8_O_3_ | 163.040 | 163.0399 | -0.8 | 91.5 | 0.5 |
| 30 | Luteoloside | 124300 | 6.82 | C_21_H_20_O_11_ | 447.093 | 447.0933 | 0.0 | 77.8 | 2.0 |
| 31 | Rutin | 34360 | 6.92 | C_27_H_30_O_16_ | 609.146 | 609.1455 | -1.0 | 98.5 | 1.9 |
| 32 | Resveratrol | 2302000 | 7.11 | C_14_H_12_O_3_ | 227.071 | 227.0713 | -0.4 | 95.5 | 0.3 |
| 33 | Polydatin | 10180000 | 7.11 | C_20_H_22_O_8_ | 389.124 | 389.1241 | -0.1 | 98.0 | 0.3 |
| 34 | Liquiritin | 524000 | 7.26 | C_21_H_22_O_9_ | 417.119 | 417.1192 | 0.3 | 98.7 | 0.7 |
| 35 | Epicatechin Gallate | 2037000 | 7.26 | C_22_H_18_O_10_ | 441.083 | 441.0828 | 0.2 | 95.4 | 0.5 |
| 36 | Acteoside; Verbascoside; Kusaginin | 42070 | 7.34 | C_29_H_36_O_15_ | 623.198 | 623.1982 | 0.1 | 95.3 | 3.6 |
| 37 | Astilbin | 337900 | 7.50 | C_21_H_22_O_11_ | 449.109 | 449.1090 | 0.1 | 88.0 | 1.1 |
| 38 | Nodakenin +HCOOH | 258200 | 8.04 | C_20_H_24_O_9_.HCOOH | 453.140 | 453.1405 | 0.6 | 90.7 | 2.2 |
| 39 | Isochlorogenic acid C | 425100 | 8.28 | C_25_H_24_O_12_ | 515.119 | 515.1198 | 0.6 | 98.1 | 1.9 |
| 40 | Rosmarinic acid | 367800 | 8.50 | C_18_H_16_O_8_ | 359.077 | 359.0772 | -0.1 | 99.6 | 0.6 |
| 41 | Hesperidin | 139700 | 8.55 | C_28_H_34_O_15_ | 609.182 | 609.1825 | 0.1 | 81.7 | 1.2 |
| 42 | Salvianolic acid A | 257100 | 8.57 | C_26_H_22_O_10_ | 493.114 | 493.1140 | -0.1 | 93.7 | 0.4 |
| 43 | Tracheloside +HCOOH | 270100 | 8.81 | C_27_H_34_O_12_.HCOOH | 595.203 | 595.2030 | -0.4 | 88.1 | 0.7 |
| 44 | Salvianolic acid B | 2968000 | 8.97 | C_36_H_30_O_16_ | 717.146 | 717.1460 | -0.2 | 83.3 | 1.8 |
| 45 | Scutellarin | 308900 | 9.01 | C_21_H_18_O_12_ | 461.073 | 461.0722 | -0.8 | 100.0 | 3.5 |
| 46 | Baicalin | 403500 | 9.26 | C_21_H_18_O_11_ | 445.078 | 445.0777 | 0.1 | 98.9 | 0.5 |
| 47 | Ononin +HCOOH | 83320 | 9.35 | C_22_H_22_O_9_.HCOOH | 475.125 | 475.1244 | -0.4 | 98.0 | 0.7 |
| 48 | Daidzein | 75850 | 9.54 | C_15_H_10_O_4_ | 253.051 | 253.0504 | -0.8 | 96.5 | 0.7 |
| 49 | Quercetin | 40580 | 10.04 | C_15_H_10_O_7_ | 301.035 | 301.0350 | -1.1 | 90.9 | 1.3 |
| 50 | Kirenol +HCOOH | 24510 | 10.30 | C_20_H_34_O_4_.HCOOH | 383.244 | 383.2436 | -0.9 | 100.0 | 0.8 |
| 51 | Harpagosid | 15400 | 10.31 | C_24_H_30_O_11_ | 493.172 | 493.1712 | -0.8 | 74.3 | 3.2 |
| 52 | Emodin-8-glucoside | 10910000 | 10.53 | C_21_H_20_O_10_ | 431.098 | 431.0985 | 0.3 | 91.9 | 0.6 |
| 53 | Wogonin 7-O-glucuronide | 107800 | 10.70 | C_22_H_20_O_11_ | 459.093 | 459.0928 | -1.1 | 94.9 | 3.1 |
| 54 | Dipsacoside B +HCOOH | 14210 | 10.95 | C_53_H_86_O_22_.HCOOH | 1119.559 | 1119.5590 | 0.0 | 93.7 | 3.7 |
| 55 | Asperosaponin Ⅵ | 74710 | 11.17 | C_47_H_76_O_18_ | 927.496 | 927.4952 | -0.8 | 90.8 | 2.6 |
| 56 | Naringenin | 34270 | 11.25 | C_15_H_12_O_5_ | 271.061 | 271.0611 | -0.4 | 96.9 | 4.3 |
| 57 | Physcion | 734500 | 11.70 | C_16_H_12_O_5_ | 283.061 | 283.0611 | -0.4 | 96.9 | 1.1 |
| 58 | Chikusetsusponin IVa | 24730 | 13.10 | C_42_H_66_O_14_ | 793.438 | 793.4376 | -0.5 | 72.8 | 3.7 |
| 59 | Glycyrrhizic acid | 1079000 | 13.18 | C_42_H_62_O_16_ | 821.397 | 821.3972 | 0.8 | 90.0 | 2.4 |
| 60 | Chrysosplenetin B | 34890 | 14.57 | C_19_H_18_O_8_ | 373.093 | 373.0928 | -0.3 | 86.8 | 1.1 |
| 61 | Emodin | 683100 | 16.44 | C_15_H_10_O_5_ | 269.046 | 269.0456 | 0.0 | 89.5 | 0.9 |
